# No effect of mitochondrial genotype on reproductive plasticity following exposure to a non-infectious pathogen challenge in female or male *Drosophila*

**DOI:** 10.1038/srep42009

**Published:** 2017-02-09

**Authors:** M. Nystrand, E. J. Cassidy, D. K. Dowling

**Affiliations:** 1 School of Biological Sciences, Monash University, Clayton, Victoria, 3800, Australia; 2 Department of Plant and Organismal Biology, University of Copenhagen, Denmark

## Abstract

Mitochondrial genetic variation shapes the expression of life-history traits associated with reproduction, development and survival, and has also been associated with the prevalence and progression of infectious bacteria and viruses in humans. The breadth of these effects on multifaceted components of health, and their link to disease susceptibility, led us to test whether variation across mitochondrial haplotypes affected reproductive success following an immune challenge in the form of a non-infectious pathogen. We test this, by challenging male and female fruit flies (*Drosophila melanogaster*), harbouring each of three distinct mitochondrial haplotypes in an otherwise standardized genetic background, to either a mix of heat-killed bacteria, or a procedural control, prior to measuring their subsequent reproductive performance. The effect of the pathogen challenge on reproductive success did not differ across mitochondrial haplotypes; thus there was no evidence that patterns of reproductive plasticity were modified by the mitochondrial genotype following a non-infectious pathogen exposure. We discuss the implications of our data, and suggest future research avenues based on these results.

It was long assumed that the genetic variation found within the mitochondrial genome would be neutral to selection^1^,^2^,^3^. This assumption was based on the rationale that selection should efficiently remove function-changing genetic variation from accumulating within the mitochondrial genome, given that the genome is haploid (and thus, all nucleotides will be constantly exposed to selection), and given that the products of this genome contribute to energy production and are therefore salient to eukaryotic life^4^,^5^. However, over the past two decades, it has become clear that mitochondrial genetic variation often contributes to the expression of traits entwined in organismal life-history, ranging from developmental rates^6^,^7^, to reproductive performance and longevity^1^,^8^,^9^,^10^,^11^. Additionally, polymorphisms within the mtDNA sequence have not only been associated with mitochondrial diseases^12^,^13^, but also with the risk of developing a range of other common late-onset diseases^14^,^15^.

In recent years, empirical evidence has been accumulating to suggest that phenotypic consequences of mitochondrial genetic variation might often be sex-specific^12^,^16^. Given the tight link between immunity, somatic development, and survival^17^, this contention is consistent with previously-reported mitochondrial genetic contributions to the expression of other key life-history traits^4^. However, specific evidence for a role of mitochondrial genetic involvement in the immune response remains sparse, and primarily comes from direct or indirect evidence in humans or mice, or from studies of cell lines, which have found associations between particular mitochondrial haplotypes and the expression patterns of nuclear genes involved in inflammation and apoptosis pathways pivotal to immune defense against invading pathogens^15^,^18^. For example, studies have linked certain mitochondrial haplotypes in humans to increased prevalence or disease progression of AIDS^19^, and other presumed auto-immune diseases such as Parkinson’s disease^20^ and Alzheimer’s^14^,^21^. Moreover, recent studies in humans have found associations between certain mtDNA haplogroups and the constitution of gut microbiomes, including infection with potentially pathogenic bacteria such as *Streptococcus*^22^. Taken together, these studies point to a role for the mitochondrial genome in regulating fitness responses in the face of stress introduced by the exposure to pathogens and parasites that are likely to be present in the natural environment.

Furthermore, a parallel set of evidence has been accumulating lately, which substantiates an evolutionary hypothesis that is often referred to as “Mother’s Curse”^23^. In theory, strict maternal inheritance of the mitochondrial genome means that the fate of mutations that accumulate within the mtDNA sequence will depend primarily on their phenotypic effects when expressed in females. This should enable the accumulation of male-harming, but female-benign, mutations in the mtDNA sequence^24^. This hypothesis has received empirical support from observations of mtDNA haplotypes that are associated with low male, but not female, fertility in hares^25^ and humans^26^. Further evidence for the hypothesis comes from recent experiments in *Drosophila melanogaster*, which have shown male-biased effects associated with mitochondrial genetic variation on fertility^27^,^28^, longevity^11^,^29^, and patterns of gene expression^29^,^30^; including identification of mtDNA mutations that confer male-specific decreases in infertility^31^,^32^,^33^,^34^.

Thus, there are emerging links between mitochondrial genetic variation, innate immune responses and life-history expression^15^,^17^,^35^, and these observations can be furthermore coupled with the recent evidence that mtDNA-mediated effects on the phenotype might be male-specific in expression, and that these effects might be particularly likely to shape the expression of male reproductive traits^16^. Hence, we therefore set out to explore whether the genetic variation found within the mitochondrial genome affects patterns of reproductive plasticity in response to a non-infectious pathogen challenge in the fruit fly *Drosophila melanogaster*, and furthermore, whether mitochondrial genetic effects on reproductive plasticity will be more pronounced in males.

To achieve this, we challenged male and female flies harbouring each of three distinct mtDNA haplotypes, with either a non-infectious pathogen challenge or a procedural control, and then measured their reproductive performance following this challenge. Using this approach, we were able to screen for gene-by-environment interactions involving mtDNA polymorphisms and the pathogen environment, on patterns of reproductive success, thus uncovering mtDNA-mediated effects on plasticity in reproductive success following the pathogen challenged. The mitochondrial haplotypes used in our study were chosen to maximize both genetic^29^ and phenotypic divergence^11^,^27^ between genetic strains, and have previously been demonstrated to influence male fertility, longevity, as well as patterns of nuclear and mitochondrial gene expression^11^,^27^,^29^,^30^.

Given that the previous studies have specifically linked mitochondrial genetic variation to components of the innate immune system and to the progression of auto-immune diseases, our specific goal of this study was to screen for interactions between the mitochondrial haplotype and the activation of host innate immune response on patterns of reproductive success in each of the sexes, without confounding effects associated with fighting an infection by alive and replicating pathogens^36^,^37^. Thus, the pathogen challenge used in our study consisted of a dose of “heat-killed”, and hence non-infectious bacteria, (Gram-negative bacterium *Escherichia coli* mixed with the Gram-positive bacterium *Micrococcus luteus*). It is well established that the maintenance and activation of immune function can be costly, and traded off against other life-history traits^17^,^38^,^39^,^40^, and that components of reproductive success have previously been shown to be sensitive to the species of heat-killed bacteria used in our study^37^,^41^,^42^, as well as to other immune elicitors (e.g. LPS)^43^,^44^. For example, an earlier study using a mix of *E. coli* and *M. luteus* recorded a trade-off between reproductive success (number of eggs) and the activation of a component of immunity (diptericin expression) in *D. melanogaster*^41^. Similar effects have been recorded in other invertebrate species that were challenged with heat-killed bacteria or other “dead” immune elicitors, for example red flour beetle (*Tribolium castanaeum*)^45^, mealworm beetles (*Tenebrio molitor*)^46^, and mosquitos (*Anopheles gambiae*)^47^. Thus, our approach has a strong precedent within the field of ecological immunity.

## Results

**Figure 1:**
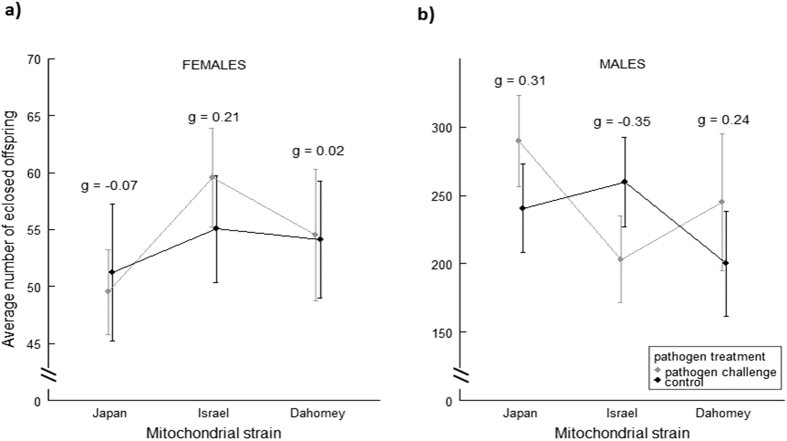
Effect of pathogen treatment and mitochondrial strain on reproductive success (cumulative mean ± SE). Numbers above SE denote effect sizes of treatment *vs*. control per haplotype. (**a**) Female reproductive success, n = [pathogen challenge/control]: Japan = [23/19], Israel = [22/23], Dahomey = [21/21]. (**b**) Male reproductive success, n = [pathogen challenge/control]: Japan = [24/22], Israel = [22/26,] Dahomey = [17/19].

Following exposure to the pathogen challenge or procedural control, the reproductive success of females and males was measured across a four day period early in life. There was no effect of the mitochondrial haplotype on the reproductive success of either sex (male reproductive success was assessed in tester females with whom they had mated), nor were there any significant interactions between haplotype and pathogen treatment on reproductive success (Table 1, Fig. 1). Thus, we find no evidence that genetic polymorphisms within the mtDNA sequence affect patterns of reproductive plasticity in females or males. Likewise, no other interactions involving mtDNA haplotype were significant (Table 1).

**Figure 2:**
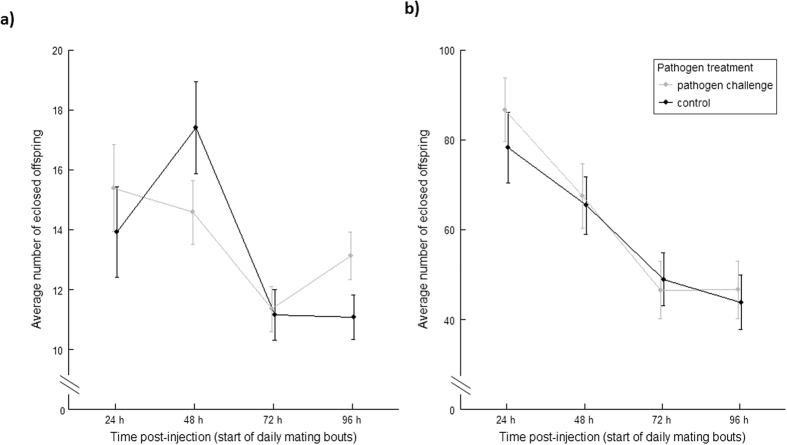
Effect of pathogen treatment on reproductive success across four days of ovipositing (X ± SE). Note that the focal females (**a**) and males (**b**) are increasing in age across the four day mating assay, whereas the tester females and males are kept at a standardized (3–4 d) age throughout. (**a**) Female reproductive success, N_individuals_ = [pathogen challenge/control]: [66/63]. (**b**) Male reproductive success, N_individuals_ = [pathogen challenge/control]: [63/67].

**Figure 3:**
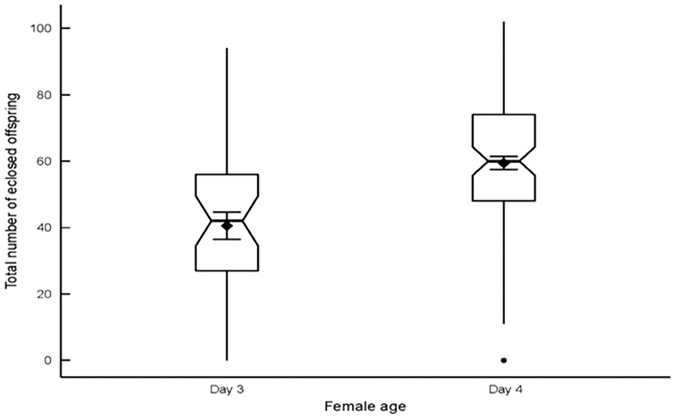
Effect of female age of mating (three days *vs*. four days) on total number of eclosed offspring (means [diamond symbol] ± SE displayed inside box plots).

The pathogen challenge *per se*, however, imposed an initial cost on female reproductive success, with pathogen-challenged females exhibiting lower reproductive output around the 48 h time point following the pathogen challenge, relative to females that received the procedural control (Table 1a, Fig. 2a). However, pathogen-challenged females subsequently compensated for this negative effect by producing more offspring than their control counterparts, in the 72 to 96 h period following treatment (Table 1a, Fig. 2a). There was also an effect of female age at mating on reproductive output (Table 1a, Fig. 3), with one day older females producing slightly more offspring.

In the male reproductive assay, there was a progressive reduction in reproductive success across the first 72 h of the reproductive assay (Fig. 2b). This decline is likely to have been caused by sperm limitation (given that the egg laying ‘tester’ females used were all of controlled age [3–4 d old] across the four days of the experiment).

Post-injection mortality (across the four days sampled) was invariably low and did not vary across the pathogen treatment in females (N treatment/control: N_Jap_ = 1/1, N_Isr_ = 1/3, N_Dah_ = 0/2; Fisher Exact test, p = 1.0) or in males (N treatment/control: N_Jap_ = 2/2, N_Isr_ = 5/3, N_Dah_ = 4/5; Fishers Exact test, p = 0.85).

Effect sizes (Hedges’ g), comparing means in pathogen-challenged versus control groups, across haplotypes, were invariably small for females and males (0 to 0.35; Table 2, Fig. 1).

## Discussion

In recent years, the results of several studies have suggested that particular mitochondrial haplotypes are associated with an increased risk of developing certain infectious and auto-immune diseases in humans^19^,^20^,^21^,^22^,^48^, and that the mitochondria might play a role in the regulation of antibacterial and antiviral responses^22^,^49^. For example, humans harbouring the UK mtDNA haplogroup have been associated with higher abundances of *Streptococcus* than humans with other haplogroups^22^. Likewise, variation in gut microbiome profiles has been shown to correlate with mitochondrial haplogroup^22^. These associations are intriguing, given that it was traditionally thought that the function of the genes encoded by the mtDNA sequence was limited to the regulation of cellular respiration^4^,^50^.

Our study constitutes an effort to complement such association analyses conducted in humans, in that we experimentally screened for interactions between the mtDNA genotype and the pathogen environment on the expression of reproductive success in fruit flies, utilising a design in which we were able to fully disentangle mitochondrial genetic from nuclear genetic effects. To achieve this, we harnessed experimentally engineered and replicated strains, in which mtDNA haplotypes were placed alongside a standardized background. We, however, found no evidence for gene-by-environment interactions involving mtDNA haplotypes and the pathogen treatment in either sex, and effects sizes across haplotypes were generally small. This suggests that genetic variation harboured within the mitochondrial genome does not interact with components of the innate immune response to perceived bacterial infection, to affect the outcomes of reproduction, at least not across the haplotypes screened here, or using the immune elicitor employed here, in this species.

In this experiment, we limited our assay to three mitochondrial haplotypes that had previously been shown to diverge molecularly (in terms of number of SNPs separating each pair of haplotypes), and phenotypically for longevity and reproductive success^11^,^27^,^29^. However, we detected no effects of mtDNA haplotype on the reproductive performance of males or females. While the lack of effect in females can be reconciled by the prediction that purifying selection should remove non-neutral mtDNA polymorphisms that affect female function^16^,^24^, the lack of an effect in males is surprising. This is because maternal transmission of mtDNA should in theory lead to the build-up of male harming (but female benign) mtDNA mutations^16^,^24^, and prior evidence has indicated male-biased mtDNA mutations typically affect components of male reproductive performance^25^,^27^,^30^,^32^. On this, we note that Yee *et al*.^28^ showed larger mitochondrial genetic effects on male fertility when fertility was measured in the presence of sperm competition with rival males, than when fertility was measured in the absence of inter-male competition. Thus, the lack of a recorded mtDNA-mediated effect on male reproductive success in our study, may stem from the nature of our design in which reproductive success was assayed in a non-competitive setting.

Our results show that the direct costs to female reproductive success, imposed by the pathogen challenge, were temporary in nature, primarily affecting females during the first 48 h post-treatment prior to a compensatory effect starting after the 72 h mark. The magnitude of this decrease in female reproductive success, and subsequent compensation, was however not contingent on the mitochondrial haplotype. This compensatory effect likely reflects the nature of the pathogen challenge, in which the pathogens (*E. coli and M. luteus*) had been heat-killed prior to its use. This was our intention – to induce costs to the host brought about by upregulation of the innate immune system *per se*, rather than costs brought about by pathogenic effects to the host of harbouring an infectious and replicating pathogen. While our results here suggest that the direct negative effects of this pathogen treatment on female reproductive success were short-lived, previous studies using very similar approaches have recently documented effects associated with exposure to a heat-killed pathogen challenge that extended across generations and shaped the expression of offspring reproductive success^37^,^42^.

We note the possibility that negative effects associated with the pathogen challenge might have affected the mating rates of flies across the treatment (pathogen challenged *versus* procedural control), potentially inflating or confounding the observed differences between treatments. While we were unable to track mating rates across the experiment associated with the pathogen treatment and control, we were able to assess whether the pathogen-challenged individuals were more likely not to have mated at all during the experiment relative to the control-treated individuals. Indeed, our data did not show any biases in the number of zero reproductive events in flies subjected to the pathogen treatment relative to flies subjected to the control (total number of individuals generating zero offspring: N_pathogen challenge_ = 5; N_control_ = 3, Odds ratio = 1.80, p = 0.49; also see Supplementary material Table S2 for a breakdown across days). These results suggest that the pathogen-treatment did not impede the capacity of treated flies to copulate and produce fertile offspring. We do, however, acknowledge that the pathogen treatment might have altered the remating behaviour of females (i.e. remating rates over and above the one mating per female), potentially affecting reproductive outcomes. While it was traditionally thought that female *D. melanogaster* enter a strong refractory period following mating that lasts up to 24 hr^51^,^52^,^53^, some studies have shown that many females will mate a second time when exposed to males following a shorter period^54^, with one study reporting up to 50% of females remating within six hours^55^. That said, we believe mating rates in our study were likely to have been low across both treatments, given females were exposed to low levels of mating interactions, cohabiting with just one other male in each 24 hour period of the 4 day assay. Finally, we note that the natural mating system of *D. melanogaster* is one where females mate multiply (allowing matings with the same and different males) within relatively short periods of time^54^,^55^, as was possible in our experimental design (i.e. matings with same and different mates, across a 96 h time period). Assessing the effects of the pathogen treatment in the presence of the natural mating rate is, we believe, the ecologically-relevant context in which to have measured the reproductive consequences of this treatment.

In sum, our key predictions were not supported. The genetic variation found across three distinct mitochondrial haplotypes had no effect on patterns of reproductive plasticity in response to a non-infectious pathogen challenge, in either sex. However, given the previously-reported links between mtDNA haplotypes in humans with abundances of certain pathogens^22^,^49^, and given the emerging conceptual link between the mitochondria and the innate immune system^22^,^49^, we suggest that further tests of the capacity for the mtDNA haplotype to influence the ability of an individual to withstand or tolerate exposure to environmental pathogens, are warranted. Such tests should use an experimental framework that has the power to unambiguously partition mitochondrial genetics from nuclear genetic effects, as employed here. We believe that the next step should be to also include a challenge involving a live pathogen, to examine the interplay between the mitochondrial genotype and the pathogen environment, over and above effects attributable to those of activation and upregulation of the host immune system *per se*, on a range of parameters including proximate (immune parameter) and ultimate (longevity, reproductive fitness) traits. Furthermore, it will be worthwhile investigating the role of epistatic interactions between mitochondrial and nuclear genetic polymorphisms, in moderating such effects, given that we know that almost all components of mitochondrial function rely on coordinated interactions between genes that span mitochondrial and nuclear genomes^50^,^56^.

## Methods

### Mitochondrial fly strains

The focal flies were sourced from three different strains, each of which harboured a different mitochondrial haplotype, sourced originally from Japan, Israel, and Dahomey. These strains are regularly screened at diagnostic markers to ensure their mitochondrial genetic identity is maintained, without contamination. The mitochondrial haplotypes were chosen to maximize levels of genetic divergence (minimum pairwise divergence between haplotypes is 18 SNPs, Supplementary material Table S1^29^), and phenotypic divergence (based on previously reported effects on male fertility and longevity between each^11^,^27^).

Each mitochondrial haplotype had been extracted and placed in an isogenic nuclear background, *w*^*1118*^ (Bloomington stock no. 5905, isogenic for chromosome 1, 2, and 3, and constructed by John Roote, Cambridge, UK) by David Clancy in 2004^57^. Upon arrival in our lab in 2007, each of these mitochondrial strains was split into two independent duplicates, and then virgin females of each mitochondrial strain duplicate were backcrossed to males of the *w*^*1118*^ strain for a further 75 generations. Isogenicity of the nuclear background was guaranteed by propagating the *w*^*1118*^ strain via mating one pair of full-siblings each generation^11^. All strains were treated, and later screened, for *Wolbachia* to ensure that they were free of infection^57^,^58^. Flies were reared under a 12:12 h light/dark cycle, at 25 °C, on a potato-dextrose-agar-medium^59^ and with *ad libitum* live yeast, at controlled egg densities (40 eggs per vial). All populations were propagated using four days old females.

### Experimental procedure

Between 20 and 25 virgins of each sex were collected per duplicate of each mitochondrial strain, and stored across two vials containing 10 flies each. These were the focal flies in the experiment. We also collected virgin flies of standardized age (3–4 days old), of each sex, from the *w*^*1118*^ isogenic strain, to be used as ‘tester’ flies that mated with focal flies during the experiment. When focal flies were three-to-four days old, females (N_3day_ = 37, N_4day_ = 92 across all mtDNA haplotypes) and males (N_3day_ = 45, N_4day_ = 85 across all mtDNA haplotypes) were subjected to either a pathogen challenge or procedural control, administered by microinjection into the abdomen (using the nano-injector “Nanoject”, Drummond Scientific Company, Broomall, PA, USA). The control consisted of 41.4 nl of PBS (Sigma Aldrich tablet P4417, pH 7.4), and the pathogen challenge consisted of 41.4 nl of a mix of equal volume heat-killed Gram-negative (*Escherichia coli*, strain K12, OD600 = 1.0, corresponding to ~27.5 × 10^6^ CFU per fly) and heat-killed Gram-positive (*Micrococcus luteus strain*, A204, OD600 = 0.1, corresponding to ~1.1 × 10^6^ CFU per fly) bacteria re-suspended in PBS (supplied by *Micromon Genomics, biotechnology and diagnostics*, Monash University). In brief, a single colony of *E. coli* and *M. luteus* had been inoculated in Nutrient broth, and incubated at 37 °C, followed by overnight shaking (105 r.p.m.). The resulting cultures were used to inoculate Nutrient broths again, followed by shaking (at 37 °C, 150 r.p.m.) until reaching the desired concentrations, upon which cell pellets were washed once in PBS and harvested by centrifugation. Cell pellets were re-suspended in PBS, and viability checks conducted. Finally, bacteria were heat-killed at 60 °C for 1 h for the *E. coli*, and 4 h for *M. luteus*, which was verified successful by bacterial growth test on Nutrient agar. Final OD and volumes were verified again before the onset of the experiment, using a spectrophotometer (UV/VIS SP8001, Metertech inc., Taiwan), and using starting volumes of 2 ml for the *E. coli* and 1.2 ml for the *M. luteus*. Pathogen treatments were administered under CO_2_ sedation, which was never administered for more than a maximum of three minutes per fly.

The aim of using a treatment consisting of a mix of Gram-positive and Gram-negative bacteria was to stimulate both main pathways of the *Drosophila* immune system; Gram-negative bacteria primarily activates the *Imd* immune pathway and Gram-positive bacteria primarily the *Toll* pathway^36^,^60^. Both these pathways are responsible for activating the production of antimicrobial peptides (AMPs) that are used in defense against bacteria and fungi^36^,^60^,^61^. Previous studies on *Drosophila* have demonstrated effects of both *M. luteus* and *E. coli* on gene or gene products associated with immune function^62^, and some studies have shown that these bacteria activate specific immune defense functions (e.g. phagocytosis)^60^,^63^. There are also demonstrated effects on phenotypic parameters^37^,^41^. Hence, we anticipated that the administration of a mix of Gram-positive and Gram–negative bacteria would facilitate an elevated response to the pathogen challenge, due to a greater perceived threat to survival^36^,^60^, which could possibly generate a longer lasting cost^43^.

Moreover, by using a heat-killed pathogen challenge rather than a live pathogen, we ensured any response to the treatment could be primarily traced to the host response *per se*, and not the indirect effects of pathogenesis induced by replicating pathogens^37^,^44^. Previous pilot experiments have indicated that the doses used for the pathogen challenge decreased reproductive success of *Drosophila* females (Supplementary Material, Fig. S1), and previous experiments identified transgenerational fitness effects tied to a challenge with *M. luteus*^37^ and by a mix *of M. luteus* and *E. coli* in fruit flies^42^.

**Figure 4:**
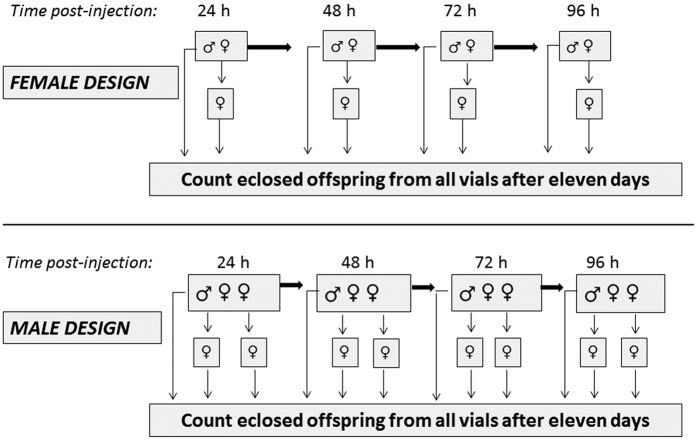
Outline of experimental design for both sexes. Females were transferred to new ovipositing vials every 24 h, each of which was retained for eleven days, at which point eclosing offspring were counted.

Following the microinjections, flies were given 24 h recovery time during which time they cohabited with other flies of the same sex in shared vials (mean: 4.4 ± 1.5 S.E. per vial), after which time they were each placed in separate vials and subjected to a four day mating trial (i.e. Day 1 covering the 24–48 h time interval post-injection [referred to as 24 h mark in graphs based on the start of the interval], Day 2 covering the 48–72 h time interval post-injection [referred to as 48 h mark in graphs], and so forth), as outlined in Fig. 4, and described below.

Focal females of each mitochondrial strain duplicate were transferred to a new vial each day for four consecutive 24 h periods, to allow ovipositing. A virgin ‘tester’ male (from the *w*^*1118*^ *strain*) of standardized age (3–4 days old) was provided to each focal female at each of the 4 consecutive 24 h periods, thus ensuring focal females could mate freely with four different males over the course of the assay. Furthermore, the reproductive assays of the focal females represent measures of early life reproductive success, and commenced when the focal females were three to four days of age. These assays of early life reproductive success provide good estimates of reproductive success over early to mid-life, since reproductive success of both females and males declines with age. Our previous work has indeed confirmed a strong correlation between reproductive success scored across the first four days post-mating and that scored across the first 10 days (R^2^ = 0.82, Fisher z = 1.49, n = 300, p = <0.0001)^37^. Likewise, a previous study using two of the same mitochondrial strains (but which, similarly to our assay on male reproductive success, sampled progressively aging males, but who were mated with a [new] standardized aged female each day) to those that were used, here also recorded the same pattern of declining reproductive success across ten days of egg laying^28^. Eleven days later, the number of adult offspring eclosing from each vial was counted, and thus female reproductive success was estimated as the number of offspring each female produced over a 96 h mating and ovipositing window.

Male reproductive success was recorded using a similar design, with the exception that males were given two standardized aged (3–4 d old) virgin tester females (of the *w*^*1118*^ strain) to mate with during each consecutive 24 h period. At the end of each 24 h mating bout, each female was transferred to an individual vial, and 11 d later, the number of offspring eclosing from each of these individual vials, as well as the vials in which the matings took place, were counted. Because each focal male was provided with two tester females per 24 h period, male reproductive scores are derived from the reproductive success of eight tester females, and were thus generally higher in magnitude than the reproductive scores of the focal female reproductive assays (see Fig. 4).

## Statistics

All statistical analyses were run in R 3.1.1 (R Development Core Team 2012)^64^. Data were zero-inflated, and models assuming a Poisson distribution over-dispersed. Zero-inflation of data was verified both graphically, by conducting a Vuong-test of fixed effects, and by conducting simulations of 95% confidence intervals around the zero values generated by a Poisson models that were corrected for over-dispersion by adding an observation-level random effect. The latter generated 2.5% and 97.5% confidence intervals (CI) of 4–17 in females, and CI of 73–125 in males. Both cases displayed a total number of zeros outside these confidence interval ranges (67 out of 516 data points in females, and 159 out of 520 data points in males). The zero-inflated models adopted here allow for the zero data to be a mix of structural and sampling zeros, thus accounting for zero values that originated from females that mated but failed to produce offspring, and females that may not have mated in the first instance. Hence, in both sexes, we fitted a negative binomial distribution to a zero-inflated model. However, in females, the NB2 fit (variance = μ(1 + μ∕k) was more appropriate, whereas in males, NB1 fit (variance = *ϕμ*) produced the better-fitting model based on AIC values^65^.

For both males and females, the response variable was the number of offspring produced, and fixed factors were the pathogen treatment (challenge, control), mtDNA haplotype (Japan, Israel, Dahomey), female age at mating (3 and 4 d), and day of ovipositing (i.e. focal females, or tester females in the male assay). While the interaction of key interest was between the pathogen treatment and mtDNA haplotype (since this would allow us to screen for mtDNA-mediated effects on reproductive plasticity), we explored all possible interactions up to first order. Random effects were the strain duplicate nested within mitochondrial strain, the vial identity nested within mitochondrial strain duplicate, and individual identity.

We also explored interactions between random effects and fixed effects. However, in no cases did random × fixed effect interactions significantly contribute to the models, and as a result, they were excluded from the analysis. Models were evaluated by removing one parameter at a time from the full model, and assessing the associated change in deviance using log-likelihood ratio tests. Model simplification then proceeded by sequentially dropping the non-significant terms (two-tailed test, α = 0.05) that had the least effect on the model, and using the resulting AIC values of all possible models to select the best-fitting model (best model highlighted in bold in Table 1).

## Additional Information

**How to cite this article**: Nystrand, M. *et al*. No effect of mitochondrial genotype on reproductive plasticity following exposure to a non-infectious pathogen challenge in female or male *Drosophila. Sci. Rep.* **7**, 42009; doi: 10.1038/srep42009 (2017).

**Publisher's note:** Springer Nature remains neutral with regard to jurisdictional claims in published maps and institutional affiliations.

## Supplementary Material

Supplementary Information

## Figures and Tables

**Table 1:** Sources of variance on total offspring production of (a) females and (b) males.

(a) Fixed effects	LRT	Pr (>χ2)
**Pathogen treatment**	**1**.**32**	**0**.**2506**
**Mitochondrial strain**	**1**.**86**	**0**.**3946**
**Female age**	**5**.**62**	**0**.**0178**
**Day of focal female ovipositing**	**84**.**7**	**<0**.**001**
Pathogen treatment × Mitochondrial strain	0.96	0.6188
Pathogen treatment × Female age	0.20	0.6547
**Pathogen treatment × Day of focal female ovipositing**	**11**.**8**	**0**.**0080**
Mitochondrial strain × Female age	2.68	0.2618
**Mitochondrial strain × Day of focal female ovipositing**	**10**.**2**	**0**.**1133**
Female age × Day of focal female ovipositing	4.28	0.2328
**Random effects (full model)**	**Variance**	
Individual	0.05241	
Duplicate (Mitochondrial strain)	2.192e-09	
Vial (Duplicate and Mitochondrial strain)	7.383e-09	
**(b)**		
Pathogen treatment	0.14	0.7083
Mitochondrial strain	1.80	0.4066
Age of the tester female	0.04	0.8415
**Day of tester female ovipositing**	**59**.**1**	**<0**.**001**
Pathogen treatment × Mitochondrial strain	3.72	0.1557
Pathogen treatment × Age of the tester female	0.06	0.8650
Pathogen treatment × Day of tester female ovipositing	1.90	0.5934
Mitochondrial strain × Female age	1.50	0.4724
Mitochondrial strain × Day of tester female ovipositing	3.32	0.7677
Tester female age × Day of tester female ovipositing	2.18	0.5359
**Random effects (full model)**	**Variance**	
Individual	0.04034	
Duplicate (Mitochondrial strain)	2.074e-09	
Vial (Duplicate and Mitochondrial strain)	2.061e-09	

Best model based on AIC comparisons of all possible models is emboldened.

**Table 2:** Effect sizes (Hedges’ g) and associated confidence intervals for the pathogen treatment (pathogen challenge and control) for each mitochondrial strain and sex.

Mitochondrial strain	sex	Hedges’ g	CI_lower_	CI_upper_	N (pathogen challenge/control)
Japan	F	0.31	−0.28	0.89	24/22
Israel	F	−0.35	−0.92	0.22	22/26
Dahomey	F	0.24	−0.42	0.89	17/19
Japan	M	−0.07	−0.65	0.50	23/23
Israel	M	0.21	−0.42	0.83	19/21
Dahomey	M	0.02	−0.58	0.61	22/21
