# Supplementary material for: No effect of mitochondrial genotype on reproductive plasticity following exposure to a non-infectious pathogen challenge in female or male Drosophila
Source: Sci Rep. 2017 Feb 9;7:42009. doi: 10.1038/srep42009 (PMC5299430; doi:10.1038/srep42009)
Supplement: Supplementary Information [file srep42009-s1.pdf]

No effect of mitochondrial genotype on reproductive plasticity following exposure to a non-infectious pathogen challenge in female or male *Drosophila*

Nystrand, M*, Cassidy, EJ. & Dowling, DK

School of Biological Sciences, Monash University, Clayton, Victoria, 3800, Australia

magdalena.nystrand@monash.edu

e.cassidy@plen.ku.dk

damian.dowling@monash.edu

**** Correspondence:***

Dr Magdalena Nystrand, School of Biological Sciences, Monash University,

Clayton, Victoria, 3800, Australia

phone: +61-(0)3-9902 4346, fax: +61-(0)3-9905 5613

email: magdalena.nystrand@monash.edu

TABLE OF CONTENTS

- Additional Tables and Figures:

  1. Table S1. Pairwise differences in number of single nucleotide polymorphisms (SNPs) between mitochondrial strains.
  2. Table S2. Frequency table showing number of zero reproductive success per day in females.
  3. Figure S1 a-d. Results of pilot experiments testing the effect of different concentrations of two different heat-killed pathogens, including procedural controls (PBS) and naïve controls, on reproductive success. Results displayed include total data averaged across treatments and the same data broken down across the four first days of sampling.

- References

ADDITIONAL TABLES AND FIGURES

Table S1. Minimum pairwise differences in number of single nucleotide polymorphisms (SNPs) delineating the three mtDNA haplotypes that comprise the mitochondrial strains. Above the diagonal are the non-synonymous SNPs, and below the diagonal are the synonymous SNPs.

	**DAH**	**ISRI**	**JAP**
**DAH**	-	8	4
**ISRI**	38	-	6
**JAP**	14	41	-

Table S2. Number of females in each treatment (pathogen challenge and control), during each reproductive event (i.e. day 1-4) that generated zero offspring (yellow) *vs.* those that generated offspring (grey).

Reproductive succcess	Treatment	Day 1	Day 2	Day 3	Day 4
# Reproductive output = 0	Pathogen treatment	14	4	4	3
# Reproductive output = 0	Control	19	9	7	7
# Reproductive output > 0	Pathogen treatment	52	62	62	63
# Reproductive output > 0	Control	44	54	56	56

Frequency test zeros: Fisher exact: $P = 0.8727$; Frequency reproduction > 0: $\chi^2 = 0.0624$, $P = 0.9959$.

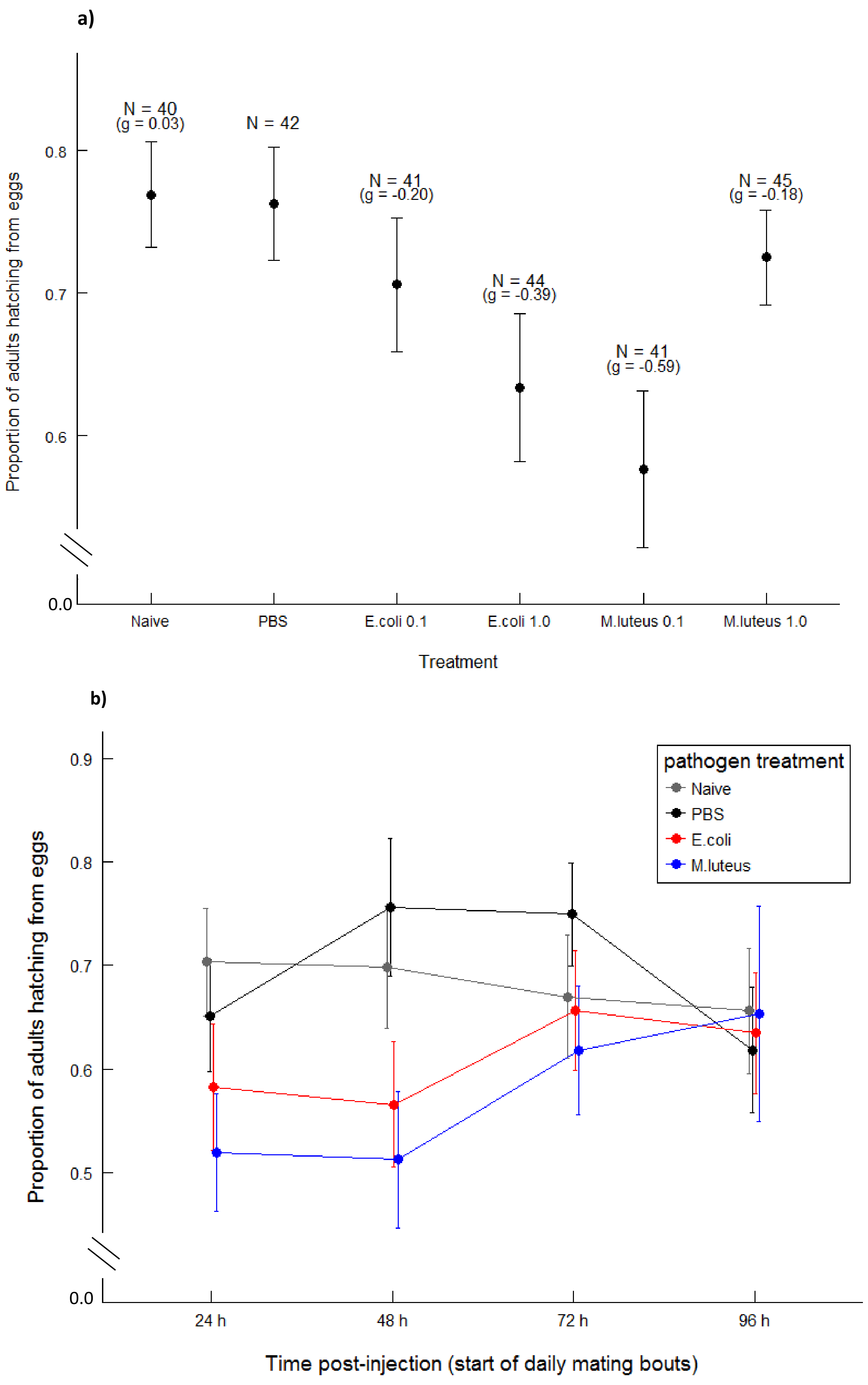
a)
Proportion of adults hatching from eggs
0.8
0.7
0.6
0.0
N = 40
(g = 0.03)
N = 42
N = 41
(g = -0.20)
N = 44
(g = -0.39)
N = 41
(g = -0.59)
N = 45
(g = -0.18)
Naive
PBS
E.coli 0.1
E.coli 1.0
M.luteus 0.1
M.luteus 1.0
Treatment
b)
Proportion of adults hatching from eggs
0.9
0.8
0.7
0.6
0.5
0.0
pathogen treatment
Naive
PBS
E.coli
M.luteus
24 h
48 h
72 h
96 h
Time post-injection (start of daily mating bouts)

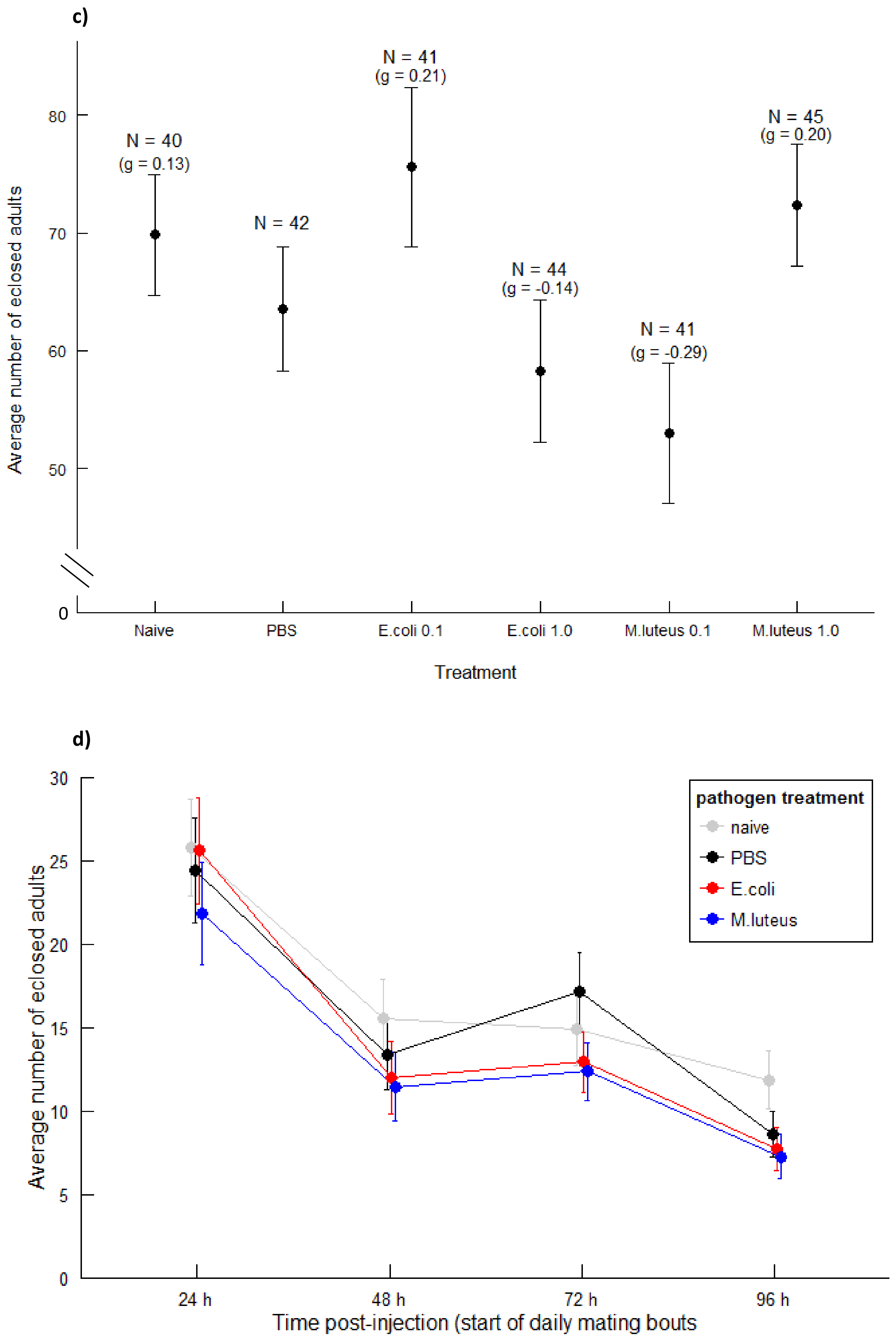
c)
N = 40
(g = 0.13)
N = 42
N = 41
(g = 0.21)
N = 44
(g = -0.14)
N = 41
(g = -0.29)
N = 45
(g = 0.20)
Average number of eclosed adults
80
70
60
50
0
Naive
PBS
E.coli 0.1
E.coli 1.0
M.luteus 0.1
M.luteus 1.0
Treatment
d)
pathogen treatment
naive
PBS
E.coli
M.luteus
Average number of eclosed adults
30
25
20
15
10
5
0
24 h
48 h
72 h
96 h
Time post-injection (start of daily mating bouts

Figure S1. Graphs displaying raw means ± SE (and for graph **a** and **c**, also including samples sizes (N) and Hedges g effect sizes (*g*) for the difference between the treatments and the procedural control, PBS). **a)** proportion adults hatching out from eggs summed across four days of sampling (showing all concentrations tested), **b)** proportion adults hatching out from eggs per day split across four days of sampling (only showing the concentrations that were selected for use on the mtDNA populations), **c)** average number of eclosed adults per treatment across the four days of sampling (all concentrations tested), and **d)** average number of eclosed adults per treatment per day across four days of sampling (only showing selected concentrations). All four graphs are generated on flies from an established lab population (Coffs population)[1-4], and show data collected post-injection by which females had been given one of the following treatments: naïve, PBS (procedural control), *E. coli* OD_{600} = 1.0, *E. coli* OD_{600} = 0.1 (only panel *a* and *c*), *M. Luteus* OD_{600} = 0.1, and *M. Luteus* OD_{600} = 1.0 (only panel *a* and *c*). Note that S1a and b reflects a scenario of density-controlled reproductive success, i.e. both these graphs display the number of adults in relation to the number of eggs laid per female. In contrast, S1c and d is not density controlled, but rather, displays the total number of adults averaged across treatments (regardless of number of eggs laid). **Statistical output** for **S1a** (*egg-to-adult*); treatment: GLM, quasibinomial error, dev = 303.11, p = 0.041 (excluding the concentrations that were not used: dev = -265.15, F = 3.079, p = 0.029). **S1b** (*egg-to-adult*); GLMM, binomial error (adjusted for overdispersion), treatment × day: dev = 12.39, p = 0.19, day: dev = 3.53., p = 0.32, and treatment: dev = 9.99., p = 0.02. **S1c** (*total adults*): GLMM, negative binomial error (NB1), treatment: dev = 13.86, p < 0.020 (and if the concentrations that were not used are excluded: dev = 9.852, p < 0.020). **S1d** (*total adults*): GLMM, negative binomial error (NB1), treatment × day: dev = 6.72, p = 0.67, day: dev = 81.58., p = < 0.001, and treatment: dev = 2.82., p = 0.42. Graphs in panel a) and c) were modified from previous work published by the authors (Nystrand, M., Cassidy, E., and Dowling, D.) which was published in BMC Evolutionary Biology (16(1), 171: DOI: 10.1186/s12862-016-0737-6), under the Creative commons Attribution 4.0 International license (http://creativecommons.org/licenses/by/4.0/).

REFERENCES

1 Williams, B. R., Heerwaarden, B., Dowling, D. K. & SgrÒ, C. M. A multivariate test of evolutionary constraints for thermal tolerance in Drosophila melanogaster. *J Evol. Biol.* **25**, doi:10.1111/j.1420-9101.2012.02536.x (2012).

2 Nystrand, M., Cassidy, E. J. & Dowling, D. K. Transgenerational plasticity following a dual pathogen and stress challenge in fruit flies. *BMC Evol. Biol.* **16**, 1-11, doi:10.1186/s12862-016-0737-6 (2016).

3 Nystrand, M. & Dowling, D. K. Dose-dependent effects of an immune challenge at both ultimate and proximate levels in Drosophila melanogaster. *J. Evol. Biol.* **27**, 876-888, doi:10.1111/jeb.12364 (2014).

4 Nystrand, M. & Dowling, D. K. Transgenerational interactions involving parental age and immune status affect female reproductive success in Drosophila melanogaster. *Proc. R. Soc. Lond., Ser. B: Biol. Sci.* **281**, doi:10.1098/rspb.2014.1242 (2014).
